# Antioxidant Profile of *Trifolium pratense* L

**DOI:** 10.3390/molecules170911156

**Published:** 2012-09-18

**Authors:** Biljana Kaurinovic, Mira Popovic, Sanja Vlaisavljevic, Heidy Schwartsova, Mirjana Vojinovic-Miloradov

**Affiliations:** 1 Department of Chemistry, Biochemistry and Environmental Protection, Faculty of Sciences, Trg Dositeja Obradovica 3, Novi Sad 21000, Serbia; Email: mira.popovic@dh.uns.ac.rs (M.P.); sanjavlaisavljevic@dh.uns.ac.rs (S.V.); 2 Middle European High School, Kráľovská 386/11, Skalica 90901, Slovakia; Email: riaditel@sevs.sk; 3 Department of Environmental Protection, Faculty of Technical Sciences, Trg Dositeja Obradovica 3, Novi Sad 21000, Serbia; Email: miloradov@uns.ac.rs

**Keywords:** *Trifolium pratense*, antioxidant activity, flavonoids, biochemical parameters

## Abstract

In order to examine the antioxidant properties of five different extracts of *Trifolium pratense* L. (Leguminosae) leaves, various assays which measure free radical scavenging ability were carried out: 1,1-diphenyl-2-picrylhydrazyl, hydroxyl, superoxide anion and nitric oxide radical scavenger capacity tests and lipid peroxidation assay. In all of the tests, only the H_2_O and (to some extent) the EtOAc extracts showed a potent antioxidant effect compared with BHT and BHA, well-known synthetic antioxidants. In addition, *in vivo* experiments were conducted with antioxidant systems (activities of GSHPx, GSHR, Px, CAT, XOD, GSH content and intensity of LPx) in liver homogenate and blood of mice after their treatment with extracts of *T. pratense* leaves, or in combination with CCl_4_. Besides, in the extracts examined the total phenolic and flavonoid amounts were also determined, together with presence of the selected flavonoids: quercetin, luteolin, apigenin, naringenin and kaempferol, which were studied using a HPLC-DAD technique. HPLC-DAD analysis showed a noticeable content of natural products according to which the examined *Trifolium pratense* species could well be regarded as a promising new source of bioactive natural compounds, which can be used both as a food supplement and a remedy.

## 1. Introduction

The Fabaceae or Leguminosae is a large and economically important family of flowering plants. The group is the third largest land plant family, with 730 genera and over 19,400 species [1]. Plants of this family are found throughout the World, growing in many different environments and climates. The *Trifolium* taxa is one of the most important genera of the Leguminosae family, both in terms of its agricultural value and the number of species (about 300) [2]. The Mediterranean region is very rich in *Trifolium* species, especially in Turkey, where it is widely spread and represented by 103 species [3]. *Trifolium pratense* L. (red clover) contains high concentrations of isoflavonoids, compounds widely distributed in the Leguminosae family [4,5]. The main isoflavones in red clover are biohanin A and formononetin [6]. Other isoflavones found in leaves include daizdein, genistein, pratensein, prunetin, pseudobaptigenin, calycosin, methylorobol, afrormosin, texasin, irilin B and irilone [7,8] and flavonoids (for example, quercetin and kaempferol). Furthermore, red clover is characterized by the presence of the other important plant phenolic substances such as phenolic acids (caffeic, rosmarinic and chlorogenic acid) [9]. Some *Trifolium* species exhibited biologically activities including anti-inflammatory activity, antioxidant activity, anticestodal activity, cytostatic activity, cytotoxic activity and estrogenic activity and are used as a chemoprotective agent against cancers and cardiovascular diseases in some traditional medicinal applications [10,11,12]. Extracts of *T. pratense* are becoming increasingly popular, primarily for the treatment of menopausal symptoms [2,13,14]. Furthermore, phytoestrogens present in *T. pratense* are also effective antioxidants and may have tyrosine kinase inhibitory activity. The antioxidant properties of genistein and other phytoestrogens have been demonstrated in several models such as protection from phorbol ester-induced singlet oxygen or peroxide formation and particularly from UV-radiation-induced oxidative damage to DNA *in vitro* [15,16,17]. In mice dietary genistein has been shown to stimulate the endogenous antioxidants, SOD, GSHPx, GSHR and glutathione S-transferase, with the effects found mainly in small intestine and the skin [18,19]. Internally, the plant is used in the treatment of skin complaints (especially eczema and psoriasis), cancers of the breast, ovaries and lymphatic system, chronic degenerative diseases, gout whopping cough and dry coughs [20].

In this study, the antioxidant activity of these extracts was determined using tests that are based on electron transfer (neutralization of DPPH radical), neutralization of free radical species (capacity of scavenging O_2_^•^^−^, OH and NO radicals) and the potential to inhibit lipid peroxidation. The actions of the potent synthetic antioxidants BHT (butlylated hydroxytoluene) and BHA (butylated hydroxyanisole), which are often used as additives in food and in the pharmaceutical, cosmetic, and in various industrial products were also investigated and compared. Knowing that phenolic compounds are the most responsible for the antioxidant activity, the amount of total phenolic contents and content of flavonoids have also been determined. Furthermore, extracts of *T. pratense* leaves were used to study *in vivo* effects on some antioxidant systems in the experimental animal’s liver and blood-hemolysate in combination with and without carbon tetrachloride (CCl_4_), in order to prove possible antioxidative activities of these secondary medicaments.

## 2. Results and Discussion

### 2.1. Determination of Total Phenolic and Flavonoid Content

Numerous investigations of qualitative composition of plant extracts have revealed the presence of high concentration of phenols in the extracts obtained using polar solvents [21]. The extracts that display the highest antioxidant activity have the highest concentration of phenols. Phenols are very important plant constituents because they are acting as scavengers of intermediate peroxyl and alkoxyl radicals, myocardial infarction, arteriosclerosis, processes of aging, and cancer, and may prevent them, and chelating agents for metal ions which are of major importance for the initiation stage of radical reactions [22]. Furthermore, phenols have therapeutic properties on different diseases like: Alzheimer’s and Parkinson’s disease, ischemic damage, arthritisdue to their antiradical property towards ROS. One of the very productive sources of polyphenols is food and food supplements of plant origin [23]. In addition, successive extractions of *T.* *pratense* were carried out, and for further work six different concentrations of extracts are prepared. Successive extraction was performed as the extraction of antioxidant substances of different chemical structure, was achieved using solvents of different polarity. Results of the amount of total phenolic contents and content of total flavonoids in *T. pratense* extracts are given in Table 1.

**Table 1:** The amount of total phenolic contents andcontent of total flavonoids in *T. pratense* extracts.

Extract	Et_2_O	CHCl_3_	EtOAc	*n*-BuOH	H_2_O
Total phenolic content	0.22 ± 0.03	0.16 ± 0.02	0.43 ± 0.01	0.21 ± 0.03	0.34 ± 0.03
Total flavonoids	11.78 ± 0.04	9.24 ± 0.03	15.23 ± 0.01	11.87 ± 0.03	15.13 ± 0.05

Total phenolic content is expressed in mg GAE/g d.e. ± S.D; Content of total flavonoids is expressed in μg RE/g d.e. ± S.D.

The amount of total phenolics in *T. pratense* extracts ranged from 0.16 ± 0.02 mg GAE/g d.e. (CHCl_3_ extract) to 0.43 ± 0.01 mg GAE/g d.e. (EtOAc extract). A significant amount of these compounds has also been observed in the H_2_O extract (0.34 ± 0.03 mg GAE/g d.e.). Furthermore, a considerable total flavonoids content was determined in the H_2_O and EtOAc extracts. A little less amount of total flavonoids was determined in the *n*-BuOH extracts, while the smallest quantity of these compounds was found in the Et_2_O and CHCl_3_ extracts. HPLC-DAD analysis indicates a significant presence of flavonoids and phenolic in the EtOAc and H_2_O extracts. Quercetin glycosides and flavonoids (kaempferol-3-*O*-Glc, quercetin-3-*O*-Glc, luteolin-7-*O*-Glc and apigenin-7-*O*-Glc) were detected in EtOAc extract, while the presence of phenolic acids was proven in the H_2_O extract (such as caffeic acid) and flavonoids (luteolin, apigenin, naringenin and kaempferol. In addition, two phytoestrogens (daizdein and genistein) were detected in the H_2_O extract.

### 2.2. *In Vitro* Experiments

The antioxidant activity of *T. pratense* extracts has been evaluated in a series of *in vitro* tests (Table 2). The 1,1-diphenyl-2-picrylhydrazyl (α,α-diphenyl-β-picrylhydrazyl; DPPH) molecule is characterised as a stable free radical by virtue of the delocalisation of the spare electron over the molecule as a whole, so the molecules do not dimerise, as would be the case with most other free radicals. The delocalisation also gives rise to the deep violet colour, characterised by an absorption band in methanol solution centered at about 520 nm. When a solution of DPPH is mixed with that of a substance that can donate a hydrogen atom, then this gives rise to the reduced form with the loss of this violet colour (although there would be expected to be a residual pale yellow colour from the picryl group still present). In the DPPH assay, the ability of the investigated extracts to act as donors of hydrogen atoms or electrons in transformation of DPPH into its reduced form DPPH-H was investigated.

**Table 2:** IC_50_ values (μg/mL) of extracts of *T. pratense* and standards (BHT and BHA) for different antioxidant assays.

Extract	Et_2_O	CHCl_3_	EtOAc	*n*-BuOH	H_2_O	BHT	BHA
**DPPH radical**	20.36	34.19	17.81	29.47	17.47	14.31	11.08
**O_2_^•−^ radical**	55.80	92.37	20.91	28.48	10.77	10.46	8.41
**NO radical**	26.66	58.46	15.67	30.64	13.33	8.63	6.31
**OH radical**	41.66	69.30	19.79	39.21	18.44	24.12	22.17

All of the assessed extracts of *T. pratense* were able to reduce the stable, purple-colored radical DPPH to the yellow-colored DPPH-H form with IC_50_ (50% of reduction) values as follows: 17.47 μg/mL for H_2_O, 17.81 μg/mL for EtOAc, 20.36 μg/mL for Et_2_O, 29.47 μg/mL for *n*-BuOH, and 34.19 μg/mL for CHCl_3_extract. Comparison of the DPPH scavenging activity of the investigated *T. pratense* extracts with those expressed by BHT (14.31 μg/mL) and BHA (11.08 μg/mL) showed that neither of the extracts showed better antioxidant properties than the synthetic antioxidants. A high degree of correlation is observed between total phenol content and the ability of EtOAc and H_2_O extracts to neutralize DPPH radicals. This is indicated by the fact that phenolic compounds play a key role in neutralizing free radical species which occurs by the mechanism of electron transfer. A lower degree of correlation defined between the content of total flavonoids in the two extracts and neutralization of DPPH radical, indicating that the content of flavonoids affect the level of free radical neutralization, but that is not directly correlated. These results are consistent with the data that is in *T. pratense* are other types of phenolic compounds such as caffeic acid, which, in addition to flavonoids, may also participate in this type of reaction and contribute to the total antioxidant activity. When investigating neutralization of O_2_^•^^−^ and NO radicals, H_2_O extract demonstrated highest activity. However, these activities were less than that of standards BHT and BHA. The lowest antioxidant activity was expressed by the CHCl_3_ (IC_50_ = 92.37 μg/mL for O_2_^•^^−^ and IC_50_ = 58.46 μg/mL for NO radical) extract. The cellular damage resulting from hydroxyl radical (OH^•^) is strongest among free radicals. Hydroxyl radical can be generated by biochemical reaction. Superoxide radical is converted by SOD (superoxide dismutase) to H_2_O_2_, which can subsequently produce extremely reactive OH^•^ radicals in the presence of transition metal ions such as iron and cooper [24]. A good antioxidant potential of neutralization OH radical were shown by the H_2_O (IC_50_ = 18.44 μg/mL) and EtOAc (IC_50_ = 19.79 μg/mL) extracts, which showed even better ability to neutralize OH radicals than BHT and BHA (IC_50_ = 22.17 μg/mL). The worst effect on the neutralization of all the radicals studied *in vitro* was shown by the *n*-BuOH extract and this is probably due to the absence of any known “scavenger” of free radicals in that extract. GC-MS analysis showed that the *n*-BuOH extract contains flavonoids in the form of glycosides and diglycosides. From the literature it is known that additional glycosylation reduces the antioxidant activity [25]. Furthermore, it can be supposed that such antioxidant activity is caused, besides flavonoids and fenols, also by terpenoids since nonpolar solvents such as Et_2_O also exhibited high antioxidant potential for neutralization of DPPH and NO radicals.

**Figure 1:**
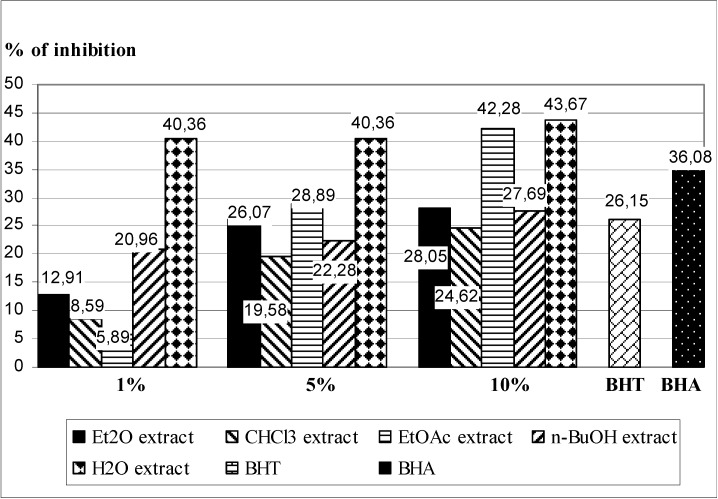
Inhibition of LP in Fe^2+^/ascorbate system of induction by by five different extracts of *T. pratense* leaves, and BHT and BHA (as a positive control) in the TBA assay.

Lipid peroxidation is an established mechanism of cellular injury and is used as an indicator of oxidative stress. Polyunsaturated fatty acids peroxides generate malondialdehyde (MDA) and 4-hydroxyalkanals upon decomposition [26]. Inhibition of LP was determined by measuring the formation of secondary components (mainly MDA) of the oxidative stress, using liposomes as an oxidizable substrate.The lipid peroxidation suppressing activity of *T. pratense* leaves extracts is shown in Figure 1, with BHT and BHA as control. In general, the examined extracts of *T. pratense* leaves expressed strong antioxidant capacity. The largest inhibitory activity, again, was exhibited by H_2_O extract. Solutions of all concentations (1%, 5% and 10%) have exhibited a stronger protective effect (from 40.36 to 43.67% of inhibition of LP) than BHT (26.15%) and BHA (36.88%). 5% and 10% solutions of EtOAc extracts demonstrate better protective effect of BHT, but only the most concentrated solution (10%) shows better inhibitory properties than BHA. The other two extracts (Et_2_O and *n*-BuOH), at the highest concentration (10%), have also exibited more intense protect effect than BHT.

Such a good antioxidant activity of H_2_O and EtOAc extracts is expected because it is known that the antioxidant activity of phenols is primarily a result of the ability of these compounds to act as donors of hydrogen atoms removing free radicals with the formation of less reactive phenoxyl radicals [27]. The increased stability of the formed phenoxyl radicals primarily attributed to electron delocalization and the existence of multiple resonant forms. Researching dependence of activity on the structure was found to have three structural features important factors of radicals removal potential and/or antioxidant potential of flavonoids: (1) *o-*dihydroxy function of ring B, which serves as the target of radicals; (2) 2,3-double bond in conjugation with 4-oxo function, which are responsible for electrons delocalization of the ring B; and (3) the additional presence of 3- and 5-hydroxyl groups for the maximum radical scavenging potential [28]. The positive relationship between increased hydroxylation and increased antioxidant activity of flavonoids was found in different lipid systems, such as oil and liposomes systems. Also, for phenolic acids and coumarins has been shown that vicinal diol groups are important for radical scavenging capacity, and that methoxylation or glycosylation of o-hydroxy group in the coumarins and esterification of phenolic acids reduce the antioxidant activity of these compounds [29]. Furthermore, the action of some flavonoids is based on their ability to chelate transition metal ions, thereby preventing the formation of radicals (initiators of LP), caught radicals initiators of LP (ROS), scavenge lipid-alkoxyl and lipid-peroxyl radicals and regenerate α-tocopherol by reduction of α-tocopheryl radicals. Different metals have different binding affinity of the flavonoids [30]. Thus, for example, iron has the highest binding affinity for 3-OH group of ring C, then catechol group ring B and at the end of 5-OH group of ring A, while the copper ions bind to the first ring catechol group B [31]. Recently, many scientists study the antioxidant properties from other *Trifolium* species. For example, Kolodziejczyk *et al.* [32] investigated the antioxidative effects of the clovamide-rich fraction, obtained from aerial parts of *Trifolium pallidum*, in the protection of blood platelets and plasma against the nitrative and oxidative damage, caused by peroxynitrite (ONOO^−^) and established that the presence of clovamide-rich *T. pallidum* extract partly inhibited ONOO^−^-mediated protein carbonylation and nitration. In addition, they determined that the *T. pallidum* extract reduced lipid peroxidation in plasma but, the antioxidative action of the tested extract in the protection of blood platelet lipids was less effective. Malinowska *et al.* [33] investigated *in vitro* oxidative changes in human plasma induced by the model of hyperhomocysteinemia in the presence of the phenolic fractions from selected clovers (*T. pallidum* and *T. scabrum*) and established that the tested phenolic fractions significantly inhibited the oxidative stress in plasma treated with homocysteine or homocysteine thiolactone. The phenolic fractions from *T. pallidum* and *T. scabrum* also caused a distinct reduction of plasma lipid peroxidation (measured by the level of thiobarbituric acid reactive substance) induced by the model of hyperhomocysteinemia.

### 2.3. *In Vivo* Experiments

The presented antioxidant activity results show that the EtOAc and H_2_O extracts of *T. pratense* leaves are efficient in protection of tissues and cells from oxidative stress. However, studies on their antioxidant status in animal model are needed to evaluate their potential health benefits. In addition, examinations of the *in vivo* activity of extracts of *T. pratense* leaves were conducted. The experimental animals were given 1 mL/kg of 2% of Et_2_O, CHCl_3_, EtOAc, *n*-BuOH or H_2_O extract (i.p.) of *T. pratense* leaves for 7 days. After 7 days, the animals were sacrificed. In the liver homogenate and blood-hemolysate of sacrificed animals the following biochemical parameters were determined: LPx intensity, content of GSH and activities of GSHPx, GSHR, Px, CAT and XOD (Table 3 and Table 4). In Table 5 and Table 6 the results of the same parameters obtained after pretreatment of experimental animals with the examined *T. pratense* extracts, followed by a single dose of carbon tetrachloride (CCl_4_) as a well-known radical generator are presented.

**Table 3:** Effect of extracts of *T. pratense* leaves on the biochemical parameters in the liver homogenate.

Parameter	Control	Et_2_O extract	CHCl_3_ extract	EtOAc extract	*n*-BuOH extract	H_2_O extract
GSH	4.17 ± 0.21	3.54 ± 0.19 ^a^	3.17 ± 0.17 ^a^	4.41 ± 0.15	3.91 ± 0.26	3.81 ± 0.23
GSHPx	5.18 ± 0.26	4.48 ± 0.19 ^a^	4.38 ± 0.21 ^a^	3.64 ± 0.25 ^a^	4.29 ± 0.16 ^a^	4.31 ± 0.25 ^a^
GSHR	6.16 ± 0.28	5.67 ± 0.24 ^a^	5.41 ± 0.26 ^a^	6.78 ± 0.17 ^a ^	5.93 ± 0.19	5.71 ± 0.22
Px	5.81 ± 0.23	5.58 ± 0.18	5.89 ± 0.21	6.17 ± 0.16	6.02 ± 0.24	6.65 ± 0.25 ^a^
LPx	7.24 ± 0.28	6.56 ± 0.91 ^a^	6.95 ± 0.16	5.34 ± 0.23 ^a^	6.41 ± 0.23 ^a^	6.19 ± 0.22 ^a ^
CAT	5.11 ± 0.19	4.78 ± 0.19	5.18 ± 0.11	6.08 ± 0.25 ^a ^	5.78 ± 0.23 ^a^	5.86 ± 0.27 ^a^
XOD	6.32 ± 0.27	6.03 ± 0.14	6.17 ± 0.26	5.38 ± 0.28 ^a^	5.43 ± 0.21 ^a^	5.12 ± 0.28 ^a^

*t*-test ^a^ *p* ≤ 0.05, n = 6, x ± SD. Content of GSH is expressed in nmol GSH/mg of protein. Activities of GSHPx, GSHR, Px, CAT and XOD are expressed in nmol/mg of protein min^−1^. Intensity of LPx is expressed in nmol malondialdehyde/mg of protein.

**Table 4:** Effect of extracts of *T. pratense* leaves on the biochemical parameters in blood hemolysate.

Parameter	Control	Et_2_O extract	CHCl_3_ extract	EtOAc extract	*n*-BuOH extract	H_2_O extract
GSH	6.17 ± 0.17	5.32 ± 0.19 ^a^	5.13 ± 0.16 ^a^	5.64 ± 0.28 ^a^	5.53 ± 0.23 ^a^	5.37 ± 0.17 ^a^
GSHPx	8.57 ± 0.24	7.31 ± 0.26 ^a^	7.14 ± 0.19 ^a^	7.87 ± 0.21 ^a^	7.77 ± 0.25 ^a^	7.51 ± 0.23 ^a^
GSHR	6.64 ± 0.18	6.81 ± 0.19	6.92 ± 0.25	7.51 ± 0.23 ^a^	7.34 ± 0.19 ^a^	7.15 ± 0.18 ^a^
Px	3.27 ± 0.19	2.54 ± 0.23 ^a^	2.79 ± 0.28 ^a^	3.14 ± 0.19	2.48 ± 0.25 ^a^	2.63 ± 0.20 ^a^
LPx	7.81 ± 0.29	7.46 ± 0.27	6.59 ± 0.31 ^a^	6.08 ± 0.23 ^a^	6.64 ± 0.33 ^a^	5.98 ± 0.26 ^a^
CAT	5.37 ± 0.27	5.87 ± 0.24	5.66 ± 0.29	6.14 ± 0.18 ^a^	6.05 ± 0.11 ^a^	5.72 ± 0.22
XOD	6.15 ± 0.29	6.62 ± 0.29	6.44 ± 0.17	5.13 ± 0.14 ^a^	5.21 ± 0.28 ^a^	5.88 ± 0.26 ^a^

*t*-test ^a^ *p* ≤ 0.05 n = 6; x ± SD. Content of GSH is expressed in μmol GSH/mL erythrocytes. Activities of GSHPx, GSHR, Px, CAT and XOD are expressed in nmol/mL erythrocytes min^−1^. Intensity of LPx is expressed in nmol malondialdehyde/mL erythrocytes.

**Table 5:** Effect of *T. pratense* leaves extracts and CCl_4_ on the liver homogenate biochemical parameters.

Parameter	Control	Et_2_O extract + CCl_4_	CHCl_3_ extract + CCl_4_	EtOAc extract + CCl_4_	*n*-BuOH extract + CCl_4_	H_2_O extract + CCl_4_
GSH	3.81 ± 0.17	2.68 ± 0.18 ^a^	2.41 ± 0.19 ^a^	3.71 ± 0.23	3.02 ± 0.17 ^a^	3.59 ± 0.21
GSHPx	4.41 ± 0.27	3.65 ± 0.21 ^a^	3.41 ± 0.18 ^a^	3.77 ± 0.30 ^a^	3.61 ± 0.22 ^a^	3.73 ± 0.18 ^a^
GSHR	5.17 ± 0.21	5.21 ± 0.28	4.16 ± 0.22 ^a^	4.31 ± 0.24 ^a^	5.26 ± 0.17	4.67 ± 0.18 ^a^
Px	4.47 ± 0.18	3.98 ± 0.16 ^a^	4.15 ± 0.15	4.86 ± 0.28	4.61 ± 0.23	4.78 ± 0.25
LPx	8.48 ± 0.26	8.13 ± 0.24	7.87 ± 0.19 ^a^	6.46 ± 0.19 ^a^	6.93 ± 0.28 ^a^	6.97 ± 0.27 ^a^
CAT	4.48 ± 0.17	3.76 ± 0.25 ^a^	3.77 ± 0.25 ^a^	4.68 ± 0.22	4.88 ± 0.21 ^a ^	4.54 ± 0.18
XOD	8.56 ± 0.28	8.89 ± 0.27	8.78 ± 0.25	8.04 ± 0.19 ^a^	7.95 ± 0.18 ^a^	8.03 ± 0.13 ^a^

*t*-test ^a^ *p* ≤ 0.05 n = 6, x ± SD. Content of GSH is expressed in nmol GSH/mg of protein. Activities of GSHPx, GSHR, Px, CAT and XOD are expressed in nmol/mg of protein min^−1^. Intensity of LPx is expressed in nmol malondialdehyde/mg of protein.

**Table 6:** Effect of extracts of *T. pratense* leaves and CCl_4_ on the biochemical parameters in blood hemolysate.

Parameter	Control	Et_2_O extract + CCl_4_	CHCl_3_ extract + CCl_4_	EtOAc extract + CCl_4_	*n*-BuOH extract + CCl_4_	H_2_O extract + CCl_4_
GSH	4.84 ± 0.27	3.26 ± 0.23 ^a^	3.87 ± 0.20 ^a^	4.27 ± 0.22 ^a^	4.05 ± 0.16 ^a^	4.34 ± 0.21 ^a^
GSHPx	7.10 ± 0.23	6.03 ± 0.21 ^a^	5.48 ± 0.29 ^a ^	6.41 ± 0.25 ^a^	6.88 ± 0.27	6.11 ± 0.26 ^a^
GSHR	5.31 ± 0.32	4.26 ± 0.28 ^a^	4.79 ± 0.34 ^a^	4.67 ± 0.17 ^a^	4.88 ± 0.34	4.69 ± 0.31 ^a^
Px	2.19 ± 0.21	1.84 ± 0.26	1.70 ± 0.18 ^a^	1.36 ± 0.13 ^a^	1.66 ± 0.18 ^a^	1.90 ± 0.21
LPx	9.23 ± 0.25	9.86 ± 0.27 ^a^	9.91 ± 0.30 ^a^	8.15 ± 0.26 ^a^	8.28 ± 0.20 ^a^	7.86 ± 0.19 ^a^
CAT	4.82 ± 0.18	4.27 ± 0.23 ^a^	3.76 ± 0.25 ^a^	4.11 ± 0.16 ^a^	4.01 ± 0.14 ^a^	4.31 ± 0.24 ^a^
XOD	8.21 ± 0.35	8.94 ± 0.27 ^a^	8.75 ± 0.28 ^a^	7.13 ± 0.18 ^a^	7.93 ± 0.17	7.38 ± 0.26 ^a^

*t*-test ^a^*p* ≤ 0.05 n = 6; x ± SD. Content of GSH is expressed in μmol GSH/mL erythrocytes. Activities of GSHPx, GSHR, Px, CAT and XOD are expressed in nmol/mL erythrocytes min^−1^. Intensity of LPx is expressed in nmol malondialdehyde/mL erythrocytes.

As can be seen from Table 3, all extracts decreased the GSH content compared with control. The Et_2_O, *n*-BuOH, H_2_O extracts, and especially the CHCl_3_ one, decreased the GSH content in the liver homogenate, whereas this value remained essentially unchanged in the treatment with EtOAc extract. The lowered GSH content in the case of treatment with the former four extracts suggests that the constituents of these extracts entered no reaction with GSH, either of the radical or conjunction type, which is they did not show a hepatoprotective effect. All the extracts produced a statistically significant decrease of GSHPx.

Treatment with the EtOAc extract yielded an increase in GSHR activity, whereas the other four extracts caused a statistically significant decrease of this enzyme, which was in agreement with the action of this enzyme on GSH. Furthermore, only H_2_O extract produced a statistically significant increase in Px activity. In addition to the very important role of peroxidase in the oxidative stress there are literature data on some other actions of peroxidases. Thus, some plant peroxidases oxidize phenols to phenoxy radicals to form polymers and enable their removal from industrial wastewaters [34]. Having in mind the results presented in Table 2, it might be interesting to test the aqueous extract of *T. pratense* as a biological marker. The LPx intensity was lowered in the liver homogenate of animals treated with all extracts of *T. pratense* leaves. The decrease was statistically significant in the case of Et_2_O, EtOAc, *n*-BuOH and H_2_O extracts, which indicates the existence of a protective effect in the *in vivo* experiments too. The CAT increased in the treatments with EtOAc, *n*-BuOH and H_2_O extracts, the other extracts caused no essential changes of CAT with respect to control. The results obtained in this assay showed a statistically significant decrease of XOD activity in the experimental animals treated with last three extracts (EtOAc, *n*-BuOH and especially H_2_O).

Summarizing the results obtained for all mice liver biochemical parameters, it can be concluded that the CCl_4_ had hepatotoxic effects, because the decreased content of GSH and also activity of GSHPx, GSHR, Px and CAT, which significantly weakened antioxidant defense system of the body (Table 5). On the other hand, it has led to an increase in the intensity of lipid peroxidation and the activity of the enzyme xanthine oxidase, which participate in the production of free radicals. It is assumed that CCl_4_ toxicity stems from the possibility of its transformation into free radicals CCl_3_^•^ and CCl_3_COO^•^, the latter of which initiates the process LP higher polyunsaturated fatty acids, which eventually leads to cell death [35]. Treatment of experimental animals with extracts and CCl_4_ generally decreased the content of GSH, probably causing the appearance of its prooxidative metabolites. Application of three *T. pratense* leaves extracts (Et_2_O, CHCl_3_ and *n*-BuOH) in combination with CCl_4_ resulted in a greater decrease of GSH content.

The administration of all examined extracts together with CCl_4_ decreased the activity of GSHPx in the liver, particularly CHCl_3_ and *n*-BuOH extracts. A combination of Et_2_O and *n*-BuOH extracts and CCl_4_ in the treatment of animals did not effect the essential change in GSHR activity. The application of CHCl_3_, EtOAc and H_2_O extracts exhibited a significant decrease of activity of this enzyme. In combination with CCl_4_ the extracts exhibited different effects on Px: while the Et_2_O extract showed a statistically significant decrease, the other four extracts had no statistically significant effect on the Px activity. All tested extracts showed inhibitory effects on LP in the liver of experimental animals, and the most active extract was EtOAc. It is assumed that the phenolic compounds present in the extract, effecting on CCl_4_-induced LP in the following ways: removing CCl_3_^•^ radicals, inhibiting microsomal Cyt P450 system (whose increased activity accelerates the transformation of CCl_4_ to free radical), removing peroxy- and lipoperoxy- radicals and complexing Fe^2+^ ions [36]. Combined treatment with the extracts and CCl_4_ had a different effect on the activity of CAT in the liver homogenate. While the Et_2_O and CHCl_3_ extracts, caused a decrease, and the *n*-BuOH extract an increase, the EtOAc and H_2_O extracts showed no effect on this parameter. The application of EtOAc, *n*-BuOH and H_2_O extracts in combination with CCl_4_ significantly lowered the activity of XOD. On the contrary, Et_2_O or CHCl_3_ extracts exhibited increasing activity on values of XOD, but statistically insignificant. Some recent studies point to the relationship between elevated XOD activity and oxidative stress in hypertension and the production of oxygen radicals in diabetes [37]. However, allopurinol, a XOD inhibitor known in clinical practice, reduces oxidative stress in diabetes [38], interacting with some peroxy radical species, such as CCl_3_OO^•^. It can be supposed that the active constituents present in EtOAc, *n*-BuOH and H_2_O extracts extracts act similarly, reducing the activity of this enzyme.

Similar to the results presented in Table 3, the results of biochemical parameters measured in blood hemolysates of animals treated with extracts of *T. pratense* leaves are shown in Table 4. Concentrations of GSH measured in blood hemolysate showed significantly lower levels after treatment of animals with all extracts. In addition, all examined extracts exhibited decreases of GSHPx values, compared with the control. 

An unusual result was obtained by examining the impact on the value of GSHR extracts. All extracts increased the activity of this enzyme, and the EtOAc, *n*-BuOH and H_2_O extract statistically significantly show this increase. As for the Px activity, all extracts reduced the activity of this enzyme, the difference being statistically insignificant only with the EtOAc extract. Four extracts, CHCl_3_, EtOAc, *n*-BuOH and H_2_O, induced a significant decrease of LPx intensity, while the Et_2_O one decreased the level of this enzyme insignificantly. On the other hand, last three extracts (EtOAc, *n*-BuOH and H_2_O) caused a statistically significant increase in CAT activity. The same extracts decreased the activity of XOD, while the other extracts caused no essential changes.

In Table 6 the results of biochemical parameters measured in the blood hemolysate of animals treated with extracts of *T. pratense* leaves and CCl_4_ are presented. As during testing of the effect of *T. pratense* leaves extracts and CCl_4_ on the liver homogenate biochemical parameters (Table 5), application of CCl_4_ caused a decrease of the GSH content and activities of GSHPx, GSHR, Px and CAT, and led to an increase in the intensity of lipid peroxidation and the activity of the enzyme xanthine oxidase. A comparasion of GSH values in Table 4 and Table 6 shows that the GSH value is significantly lower in the animals treated with CCl_4_ (6.17 ± 0.17 *vs.* 4.84 ± 0.27). This indicates that GSH acts as one of the essential antioxidant systems. On the other hand, the extracts showed no protective effect; moreover, all of them produced a further decrease of the GSH value. The administration of all extracts, except *n*-BuOH, significantly decreased the activity of GSHPx. *n*-BuOH extract did not cause notable changes. From the results presentd in Table 6, it is obvious that CCl_4_ decreased the values of GSHR (5.31 ± 0.32 nmol/mL erythrocytes x min^−1^), compared with the levels in the control group (6.64 ± 0.18 nmol/mL erythrocytes min^−1^). The results of GSHR activity were significantly lower in the combination of CCl_4_ with all extracts, except *n*-BuOH. Also, treatment with *n*-BuOH caused a decrease of GSHR activity, but not notably (4.88 ± 0.34 nmol/mL erythrocytes min^−1^).

The LPx value showed a statistically significant increase with CCl_4_-treated animals compared with the untreated ones, whereas the presence of EtOAc, *n*-BuOH and H_2_O extracts yielded a decrease of LPx. These results suggest that these three extracts had a protective effect. According to the literature data [39], the reduction of the serum LPx might be the result of antioxidant activity of several classes of plant phenolic constituents, such as cinnamic acids (ferulic, caffeic, and chlorogenic), flavonoids and biflavonoids, 1,3,6,7-tetrahydroxyxanthones, and acylphoroglucinols such as hyperforin and adhyperforin. On the other hand, the Et_2_O and CHCl_3_ extracts significantly increased the activity of LPx in combination with CCl_4_. Very indicative are the results obtained for the Px and CAT which are significantly decreased in combination of all extracts with CCl_4_. A statistically significant decrease of XOD activity was observed in the case of treatment with the EtOAc and H_2_O extracts, while *n*-BuOH extract exhibited no influence on it. Application of other two extracts (Et_2_O or CHCl_3_) with CCl_4_, expressed a statistically significant increase of XOD.

## 3. Experimental

### 3.1. Chemicals

Thiobarbituric acid (TBA), gallic acid, xanthine, xanthine-oxidase, ethylenediaminetetraacetic acid (EDTA), 2,2-diphenyl-1-picrylhydrazyl (DPPH) and trichloroacetic acid were purchased from Sigma-Aldrich Chem (Steinheim, Germany). Folin-Ciolcateu reagent was provided by Fisher Scientific (Leicestershire, UK). 2-Deoxy-D-ribose was purchased from Aldrich. *N*-(1-naphthyl)-ethylenediamine dihydrochloride (NEDA) was acquired from Merck (Darmstadt, Germany). Rutin, *tert*-butyl hydroxytoluene and *tert*-butyl-4-hydroxyanisole were obtained from Fluka AG (Buchs, Switzerland). The commercial preparation of liposomes “PRO-LIPO S” was purchased from Lucas-Meyer (Hamburg, Germany). All chemicals used were of analytical grade.

### 3.2. General

The plant specimen was collected in May 2009 from Vojvodina Province, Republic of Serbia. Plant material (100 g) was reduced to a fine powder and extracted by maceration with 80% aqueous methanol (3 L) during 72 h at room temperature. After the filtration, the solvent was evaporated *in vacuo* at 65 °C and the aqueous phase was successively extracted with four solvents of increasing polarity, namely ether (Et_2_O), chloroform (CHCl_3_), ethylacetate (EtOAc) and *n*-butanol (*n*-BuOH). The extraction was carried out until a colourless extract was obtained. The residue was the aqueous extract. All five extracts (Et_2_O, CHCl_3_, EtOAc, *n*-BuOH, and H_2_O) were evaporated to dryness and then dissolved in 50% ethanol to make 10% (w/v) solutions. These solutions, either as such or in diluted state, were used in subsequent experiments.

### 3.3. Determination of Total Phenolic and Flavonoid Content

For estimation of the total phenolic content the method reported by others [40] was performed. Extracts were used in concentration of 0.125, 0.25 and 0.5 mg/mL. Gallic acid, prepared in ten concentrations ranging from 0.50 to 100 μg/mL, was used as a standard. Thirty microlitres of each extract or standard solution, except in a blank probe when only the solvent was used, was added to 150 μL of 0.2 mol/L Folin-Ciolcateu reagent and mixed with 120 μL of sodium carbonate (7.5%) after 10 min. The mixture was incubated in the dark at room temperature for 2 h to complete the reaction. The absorbance of the resulting solution was measured at 760 nm on a UV/VIS spectrophotometer (CECIL CE2021). The phenolics concentration was determined by comparison with the standard calibration curve of gallic acid, and results are presented as a mean value of triplicate tests. The total phenol value was expressed as milligrams of gallic acid equivalents (GAE) per gram of dry extract (d.e.).

The aluminum chloride colorimetric method described by Jia *et al*. [41] was used to determine the total content of flavonoids. Test samples were prepared in concentrations 1.0, 2.0, and 4.0 mg/mL, whereas rutin solutions were prepared ranging from 0.50 to 100 μg/mL and used as a standard. Thirty microliters of extract or standard solution was diluted with 90 μL of methanol, and 6 μL of 10% aluminum chloride (substituted with distilled water in blank probe), 6 μL of 1 mol/L potassium acetate, and 170 μL of distilled water were added. Absorbance at 430 nm was determined after 30 min. All samples were made in triplicate, and mean values of flavonoid content are expressed as µg of rutin equivalents (μg RE) per gram of d.e. calculated according to the standard calibration curve.

### 3.4. *In Vitro* Experiments

The DPPH assay was performed as described before [42], following the transformation of the DPPH radical to its reduced, neutral form (DPPH-H). The samples of all extracts of *T. pratense* leaves were investigated in concentrations of 10.00–100.00 μg/mL. The same procedure was repeated with *tert*-butylated hydroxytoluene (BHT) and butylated hydroxyanisole (BHA) as a positive control. For each sample five replicates were recorded. The disappearance of DPPH was measured spectrophotometrically at 515 nm.

The capability of extracts to neutralize superoxide anion radical formed by the reduction of nitroblue tetrazolium (NBT) with NADH mediated by phenazine methosulfate (PMS) under aerobic conditions was conducted according to Nishikimi *et al.* [43]. The samples of all extracts of *T. pratense* leaves were investigated in concentrations of 10.00–100.00 μg/mL. The same procedure was repeated with BHT and BHA as a positive control. For each sample five replicates were recorded. The intensity of color was measured spectrophotometrically (λ = 550 nm).

Production of NO radicals was determined spectrophotometrically. NO radical generated from sodium-nitropruside (SNP) reacts with oxygen in water solution at a physiological pH to give nitrite ions. Concentration of nitrite anions was determined using Griess reagent [44,45]. At room temperature nitrite ions react with Griess reagent and form purple complex. The samples of all extracts of *T. pratense* leaves were investigated in concentrations of 10.00–100.00 μg/mL. The intensity of color, which is the function of the nitrite concentrations, was measured spectrophotometrically (λ = 546 nm). For each sample, five replicates were recorded. 

Scavenging capacity of the *T. pratense* leaves extracts for hydroxyl radicals was determined by monitoring the chemical degradation of 2-deoxy-D-ribose [46]. The reaction was initiated by hydroxyl radicals obtained in Fenton’s reaction [47], which yields products that react with thiobarbituric acid (TBA test). The samples of all extracts of *T. pratense* leaves were investigated in concentrations of 10.00–100.00 μg/mL. The obtained products, among which malondialdehyde (MDA) is the most important, are determined by a spectrophotometric method at 532 nm. The absorbance of the resulting solutions and the blank (with same chemicals, except sample) was recorded. Five replicates were performed for each sample. 

The extent of LP was determined by measuring the color of the adduct produced in the reaction between 2-thiobarbituric acid (TBA) and malondialdehyde (MDA), as an oxidation product in the peroxidation of membrane lipids, by the TBA assay [36,48,49]. The commercial preparation of liposomes “PRO-LIPO S” (Lucas-Meyer) pH = 5–7 was used as a model system of biological membranes. The liposomes, 225–250 nm in diameter, were obtained by dissolving the commercial preparation in demineralized water (1:10), in an ultrasonic bath. For the experiment, three concentrations of extracts of *T. pratense* leaves were prepared (10%, 5% and 1% solution). Five replicates were performed for each sample.

### 3.5. *In Vivo* Antioxidant Activity

This investigation was conducted on sexually mature White laboratory mice (both sexes), type BALB/C, with an average body weight of 20–25 grams. Animal care and all experimental procedures were conducted in accordance with the *Guide for the Care and Use of Laboratory Animal Resources*, edited by Commission of Life Sciences, National Research Council (USA), Male and female Hanover National Medical Institute (Hann NMRI). Experimental animals were bred in the vivarium at the Department of Pharmacology, Toxicology and Clinical Pharmacology, Medical Faculty, University of Novi Sad, Serbia. Animals were kept in standard plexiglass cages (room temperature 21 ± 1 °C; humidity 55 ± 1.5%, with 12 h light period). They were fed standard laboratory animals feed, produced by the Veterinary Institute in Zemun. Animals were given free access to food and fluid (water). Animals were randomly assigned into six groups, consisting of 10 animals each. The animals of control group were given intraperitoneally (i.p.) physiological solution, the animals of five experimental groups received at the same time (i.p., 1.0 mL/kg) one of 5 *T. pratense* leaves extracts. After 7 days five animals from each group were sacrificed to determine the biochemical liver and blood parameters. The remaing five animals of each group were treated (i.p.) with CCl_4_ in olive oil (1:1, 2.0 mL/kg) and sacrificed 24 h later, to determine the same biochemical parameters.

Examined biochemical parameters were measured in blood hemolysate and liver homogenate. Liver was homogenized in a Potter homogenizer with TRIS-HCl/sucrose in a ratio of 1:3 at 4 °C. Obtained homogenate was filtered. The following biochemical parameters were analyzed in blood hemolysate and liver homogenate: extent of lipid peroxidation (LPx) was determined after Buege and Aust [50], peroxidase (Px) activity was measured after Simon *et al*. [51], catalase activity (CAT) after Beers and Sizer [52]. Glutathione peroxidase (GSHPx) activity was evaluated as described by Chin *et al*. [53], glutathione reductase (GSHR) after Glatzle *et al.* [54], xanthine oxidase (XOD) after Bergmayer [55], content of reduced glutathione (GSH) after Kapetanović and Mieyal [56]. The total protein content in liver was determined after Gornall *et al*. [57].

### 3.6. Statistical Analysis

The percentage of RSC for each radical was calculated using the following equation: 


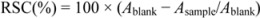


From the obtained RSC values, the IC_50_ values, which represented the concentrations of the examined extracts that caused 50% neutralization, were determined by linear regression analysis.

The percentage of LP inhibition was calculated by the following equation:


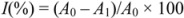


where *A*_0_ was the absorbance of the control reaction (full reaction, without the test compound) and *A*_1_ was the absorbance in the presence of the inhibitor.

Results of biochemical analyses are presented as mean value ± standard deviation (S.D.). The differences between control and test groups were analyzed using the Student *t*-test (significant difference at *p* ≤ 0.05 confidence level).

## 4. Conclusions

The presented antioxidant activity results show that the EtOAc and H_2_O extracts of *T. pratense* leaves are efficient in protection of tissues and cells from oxidative stress. Based on these results it can be concluded that the H_2_O and EtOAc extracts of leaves of *T. pratense* showed strong antioxidant activities when compared to the standards. However, the difference in the antioxidant activities of these two extracts may be due to their different phytochemical composition. The results obtained emphasize that the H_2_O and EtOAc extracts mainly exhibit their antioxidant potential via free radical scavenging and electron donation. Furthermore, a *T. pratense* leaves extracts exhibited different activities in relation to the investigated biochemical parameters. The results obtained indicate toxicity of CCl_4_, probably due to the radicals involved in its metabolism. In *in vivo* experiments the best protective effect was shown by the EtOAc and H_2_O extracts. Therefore, it seems reasonable to consider these leaf extracts as a new valuable source for pharmaceuticals in the promotion of health as commercial drugs.
